# Determination of Minimum Training Sample Size for Microarray-Based Cancer Outcome Prediction–An Empirical Assessment

**DOI:** 10.1371/journal.pone.0068579

**Published:** 2013-07-05

**Authors:** Li Shao, Xiaohui Fan, Ningtao Cheng, Leihong Wu, Yiyu Cheng

**Affiliations:** 1 Pharmaceutical Informatics Institute, School of Pharmaceutical Sciences, Zhejiang University, Hangzhou, China; 2 The Wallace H. Coulter Department of Biomedical Engineering, Georgia Institute of Technology and Emory University, Atlanta, Georgia, United States of America; 3 State Key Laboratory for Diagnosis and Treatment of Infectious Disease, First Affiliated Hospital, College of Medicine, Zhejiang University, Hangzhou, Zhejiang, China; University of North Carolina at Charlotte, United States of America

## Abstract

The promise of microarray technology in providing prediction classifiers for cancer outcome estimation has been confirmed by a number of demonstrable successes. However, the reliability of prediction results relies heavily on the accuracy of statistical parameters involved in classifiers. It cannot be reliably estimated with only a small number of training samples. Therefore, it is of vital importance to determine the minimum number of training samples and to ensure the clinical value of microarrays in cancer outcome prediction. We evaluated the impact of training sample size on model performance extensively based on 3 large-scale cancer microarray datasets provided by the second phase of MicroArray Quality Control project (MAQC-II). An SSNR-based (scale of signal-to-noise ratio) protocol was proposed in this study for minimum training sample size determination. External validation results based on another 3 cancer datasets confirmed that the SSNR-based approach could not only determine the minimum number of training samples efficiently, but also provide a valuable strategy for estimating the underlying performance of classifiers in advance. Once translated into clinical routine applications, the SSNR-based protocol would provide great convenience in microarray-based cancer outcome prediction in improving classifier reliability.

## Introduction

Recent advances in gene expression microarray technology have opened up new opportunities for better treatment of diverse diseases [1], [2], [3]. A decade of intensive research on developing prediction classifiers has yielded a number of demonstrable successes, especially the capability of predicting different potential responses to a therapy [4]. For example, it helped with treatment selection to prolong survival time and improve life quality of cancer patients. The approbation of MammaPrint™ by U.S. Food and Drug Administration (FDA) for clinical breast cancer prognosis [5] illustrated the promise of microarray technology in facilitating medical treatment in the future.

More recently, MicroArray Quality Control Project II (MAQC II) study [6] confirmed once again that microarray-based prediction models can be used to predict clinical endpoints if constructed and utilized properly. However, the reliability of prediction results relied heavily on the accuracy of statistical parameters involved in microarray classifiers, which cannot be reliably estimated from a small number of training samples. Therefore it would help by collecting as many clinical samples as possible. Nevertheless, considering the fact that relatively rare clinical tissue samples can be used for transcriptional profiling, it is a challenge to estimate an appropriate number of training samples enough to achieve significant statistical power.

Several methods have been suggested for sample size determination, such as the stopping rule [7], the power analysis algorithm [8], the parametric mixture modeling combined with parametric bootstrapping [9], sequential classification procedure based on the martingale central limit theorem [10], the parametric probability model- based methodology [11], the Monte Carlo combined with approximation approaches [12], and the algorithm based on weighted fitting of learning curves [13], etc. Most of the above studies were exploratory in nature, and focused on the relationships between sample size, meaningful difference in the mean, and power. It is rather possible for these methods to produce either an underestimated or overestimated sample size, if a specific variance and meaningful difference in the mean was used [14]. Moreover, the statistical models and/or indices utilized in above methods are quite difficult to implement in real applications, and are only feasible when enough training samples are collected. Dobbin et al. proposed a sample size calculation method based on standardized fold change, class prevalence and the number of genes or features on the arrays [15]. Although such method is quite simple compared to previous approaches, it is only adapted to address ex post facto determination of whether the sample size is adequate to develop a classifier. Thereby, a few issues have to be addressed before a simple and efficient method for sample size estimation could be developed.

Early in 2005, Van Niel et al. has pointed out that the required number of training samples should be determined by considering the complexity of the discrimination problem [16]. Standardized fold change and class prevalence proposed by Dobbin et al. are also to some extent correlated to classification complexity [15]. Popovici et al. further demonstrated that the performance of a genomic predictor is determined largely by an interplay between sample size and classification complexity [17]. In summary, figuring out the relationship between sample size, model performance, and classification complexity is of great help in developing a user-friendly sample size planning protocol.

Three large-scale microarray datasets with a total of 10 endpoints provided in MAQC-II [6] were extensively evaluated for the relationship between training sample size and the performance of constructed prediction classifiers in this study. It was found that the minimum training sample size could be estimated from the intrinsic predictability of endpoints, and we proposed an SSNR-based stepwise estimation protocol. External validation results using another three large-scale datasets confirmed the capability of this protocol. Compared to previous methods, the protocol proposed in this study has its advantages in the following three aspects: firstly, it is easier to implement and much more efficient for clinical applications; secondly, less prior information is required, and thus experimental cost could be better controlled; lastly, it guides the experimental design, in addition to the ex post facto estimation of training sample size.

## Materials and Methods

### Datasets

Six large-scale cancer datasets have been collected in this study for training sample size estimation and external validation purposes. **Table 1** illustrated a concise summary of the collected datasets, including the information about sample size and sample distribution.

**Table 1:** A concise summary of datasets.

Data Set	Endpoint Description	Endpoint Code^a^	Sample Size		Ratio of events		Microarray Platform (number of channel)
			Training	Validation	Training	Validation	
BR	Treatment Response	BR-pCR	130	100	0.34 (33/97) b	0.18 (15/85)	Affymetrix U133A (1)
		BR-erpos	130	100	1.60 (80/50)	1.56 (61/39)	
MM	Overall Survival Milestone Outcome	MM-OS	340	214	0.18 (51/289)	0.14 (27/187)	Affymetrix U133Plus2.0 (1)
	Event-free Survival Milestone Outcome	MM-EFS	340	214	0.33 (84/256)	0.19 (34/180)	
NB	Overall Survival Milestone Outcome	NB-OS	246	177	0.32 (59/187)	0.28 (39/138)	Agilent NB Customized Array (2)
	Event-free Survival Milestone Outcome	NB-EFS	246	193	0.65 (97/149)	0.75 (83/110)	
NHL	Overall Survival Milestone Outcome	NHL	160	80	1.22 (88/72)	1.67 (50/30)	Lymphochip (2)
BR2	Estrogen Receptor Status	BR2-erpos	196	90	2.70 (143/53)	2.75 (66/24)	Affymetrix U133A (1)
BR3	5-year metastasis-free survival	BR3-EFS	194	100	0.39 (54/140)	0.39 (28/72)	Affymetrix U133A (1)
Control	Positive control	NB-PC	246	231	1.44 (145/101)	1.36 (133/98)	Agilent NB Customized Array (2)
		MM-PC	340	214	1.33 (194/146)	1.89 (140/74)	Affymetrix U133Plus2.0 (1)
	Negative control	NB-NC	246	253	1.44 (145/101)	1.30 (143/110)	Agilent NB Customized Array (2)
		MM-NC	340	214	1.43 (200/140)	1.33 (122/92)	Affymetrix U133Plus2.0 (1)

a BR - Breast Cancer; MM - Multiple Myeloma; NB - Neuroblastoma; pCR - Pathologic Complete Response; erpos – ER Positive; OS – Overall Survive; EFS – Event-free Survival; NHL- non-hodgkin lymphoma; PC – Positive Control; NC – Negative Control;

b Ratio of good to poor prognoses (i.e., good/poor prognoses).

Three datasets with 10 clinical endpoints - breast cancer (BR), multiple myeloma (MM), neuroblastoma (NB), provided in MAQC-II [6] were selected and utilized in this study to evaluate the impact of training sample size on model performance. For breast cancer, endpoints BR-erpos and BR-pCR represent estrogen receptor status and the success of treatment involving chemotherapy followed by surgical resection of a tumor, respectively. For multiple myeloma, MM-EFS and MM-OS represent event-free survival and overall survival after 730 days post treatment of diagnosis, while NB-EFS and NB-OS represent the same meaning after 900 days post treatment or diagnosis. Moreover, endpoints NB-PC and MM-PC, NB-NC and MM-NC were also included in this study as positive and negative controls, respectively. The NB-PC and MM-PC were derived from the NB and MM datasets with the endpoints denoted by gender, while endpoints for NB-NC and MM-NC were generated randomly.

Another three datasets, including one non-hodgkin lymphoma (NHL) [18] dataset and two breast cancer datasets (BR2 [19] and BR3 [20]) used in previously published prognostic modeling studies, were used in this study for external validation purpose. NHL is related to the survival of non-hodgkin lymphoma [18] patients, while BR2 and BR3 are related to the estrogen receptor status (BR2-erpos) [19] and the 5-year metastasis-free survival (BR3-EFS) [20] of breast cancer patients.

To simulate the real-world clinical application of genomic studies, two independent populations of patients for each dataset created by the MAQC consortium or by the original researchers are retained in this study as the training and validation sets. In the case of BR2-erpos and BR3-EFS, there was no information for sample splitting. Thus all samples were allocated into training and validation sets randomly in this study. More detailed information about the datasets can be found in the main paper of MAQC-II [6] and its corresponding original papers.

### Statistical Analysis

**Figure 1:**
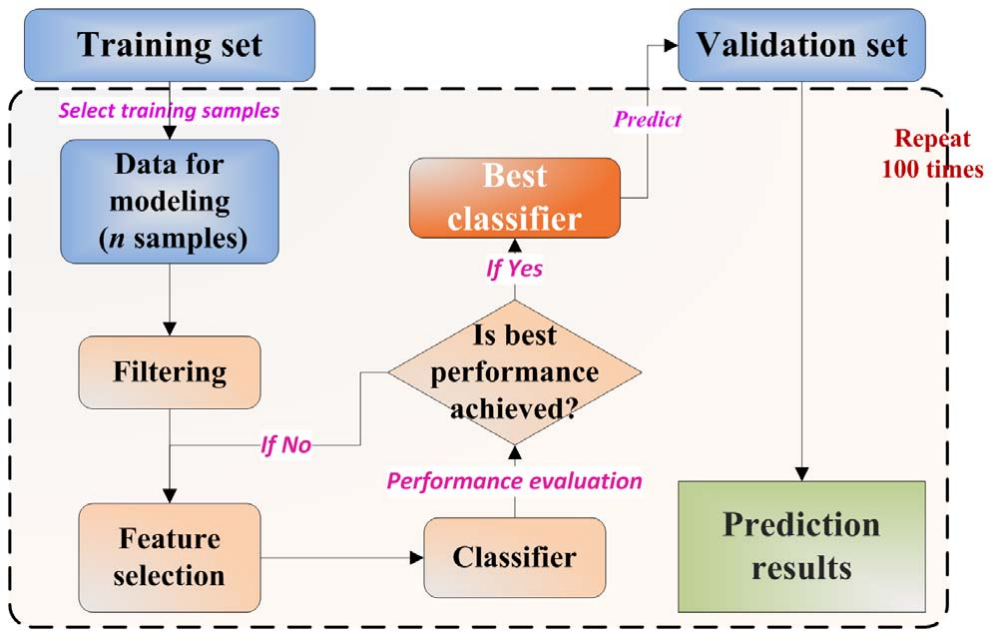
Study work flow. Work flow for evaluating the impact of different number of training samples.

Detailed information about the study design was illustrated in **Figure 1**, additional information about model construction procedure is available in Methods S1. A dataset with a specific sample size was firstly retrieved from the original training set as new training samples. After model construction from the retrieved training samples using a 5-fold cross-validation, the obtained *best classifier* was then applied to predict the original validation set. To ensure the statistical power, such procedure was repeated 100 times, resulting in 100 different sets of predictions. The average prediction result was then utilized as an indication of model performance corresponding to this specific sample size. The number of training samples considered in this study ranges from 20 with a step of 20. Three widely used machine learning algorithms including *NCentroid* (Nearest-Centroid), *kNN* (*k*-nearest neighbors, *k* = 3) and *SVM* (Support Vector Machine) were selected in this study to evaluate the impact of training sample size.

Based on the 100-run results, the trend of model performance (as measured by Matthews correlation coefficient (MCC) [21] versus the stepwise increase of training sample size is illustrated by whisker plot (5–95% percentile). The Matthews Correlation Coefficient (MCC) is defined as:
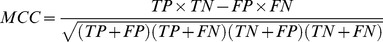
(1)where 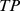 is the number of true positives, 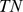 is the number of true negatives, 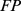 is the number of false positives and 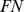 is the number of false negatives. MCC varies between −1 and +1 with 0 corresponding to random prediction.

Based on the 100-run MCC values, we further proposed an equation to approximately estimate the potential value of increasing sample size, which considers both the relative improvement of model performance and the cost of increasing sample size.
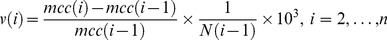
(2)


Here 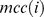 and 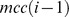 represent the MCC value obtained from the *i*th and *(i-1)*th sample size, while 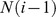 is the number of training samples at the *(i-1)*th step (*i = 2,…,n*). 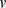 value much smaller than 1 was utilized in this study to assist in determining the near-optimal classifier. In other words, 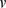 value combined with the mean and variance of MCC values was finally used to determine the near-optimal training sample size.

### Scale of Signal-to-noise Ratio (SSNR)

Suppose microarray datasets *X_1_* (*n_1_* samples and *p* genes) and *X_2_* (*n_2_* samples and *p* genes) were profiled from samples in class 1 and class 2, respectively. The signal-to-noise ratio for the *i*th gene (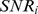, *i = 1,2,…,p*) reflects the difference between the classes relative to the standard deviations (SD) within the classes, and could be presented as follows [22]:
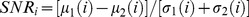
(3)


Here 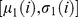 and 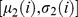 denote the means and SDs of the log of the expression levels of the *i*th (*i = 1,2,…,p*) gene in class 1 and class 2, respectively. 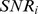
is not confined to [−1, 1], with large values of 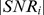 indicating a strong correlation between the gene expression and the class distinction. The sign of 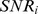 being positive and negative corresponds to the *i*th gene being more highly expressed in class 1 or class 2. SSNR is the numeric scale of 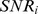 for all genes (*i = 1,2,…,p*) representing the numeric difference between the largest positive- and the smallest negative- SNR values. Assuming that 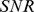 represents the vectors of SNR values for all genes in a dataset, SSNR could be defined as follows:
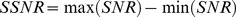
(4)


## Results

### Minimum Training Sample Size Varies with Endpoint Predictability

**Figure 2:**
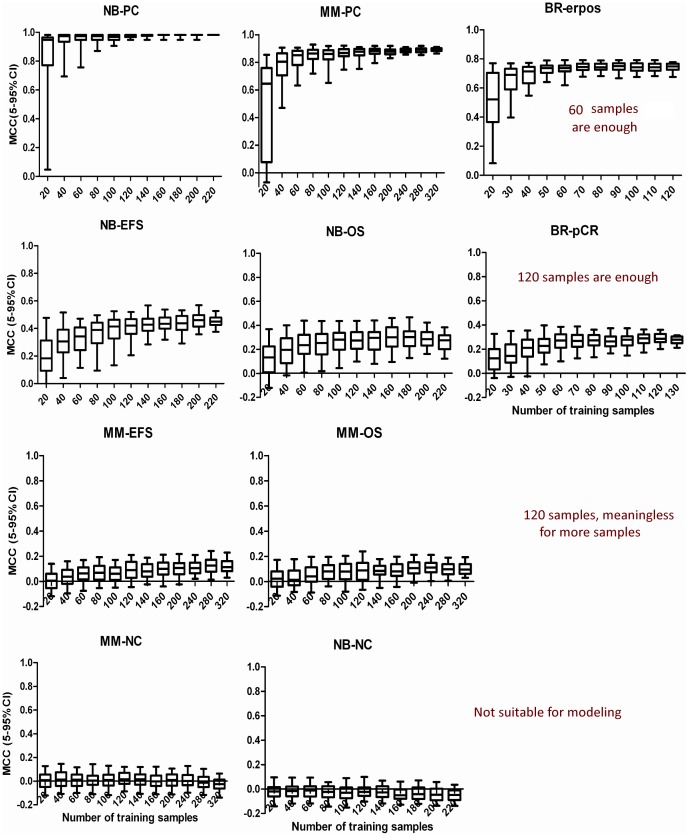
Impact of training sample size. Prediction MCC based on different number of training samples for 10 endpoints using *NCentroid*.

**Figure 2** demonstrated the trend of model performance versus stepwise increase of training sample size for 10 endpoints using *NCentroid*, with corresponding 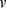 values shown in Table S1. Two conclusions can be drawn from the study. Firstly, training sample size exerted apparent effects on model performance for all endpoints except for negative controls. Secondly, the required minimum number of training samples varies with the complexity of different endpoints. For highly predictable endpoints (NB-PC, MM-PC and BR-erpos) with prediction MCC around or larger than 0.8, 60 training samples are enough to achieve near-optimal prediction classifiers. While for endpoints (NB-EFS, NB-OS, BR-pCR) with moderate prediction performance (MCC between 0.2 to 0.5), at least 120 training samples are needed. For hardly predictable endpoints (MM-EFS and MM-OS), microarray-based prediction model (MCC around 0.1) is generally not a good choice in this case. In the event when 120 samples are needed, it makes no sense to collect any more samples due to the negligible improvement. For negative controls (NB-NC and MM-NC), prediction models fail for all training sample sizes. Such results excluded the possibility of obtaining false positive results. Figures S1 and S2 obtained from *kNN* and *SVM* confirmed the above results.

### SSNR Correlates Well with Endpoint Predictability

**Figure 3:**
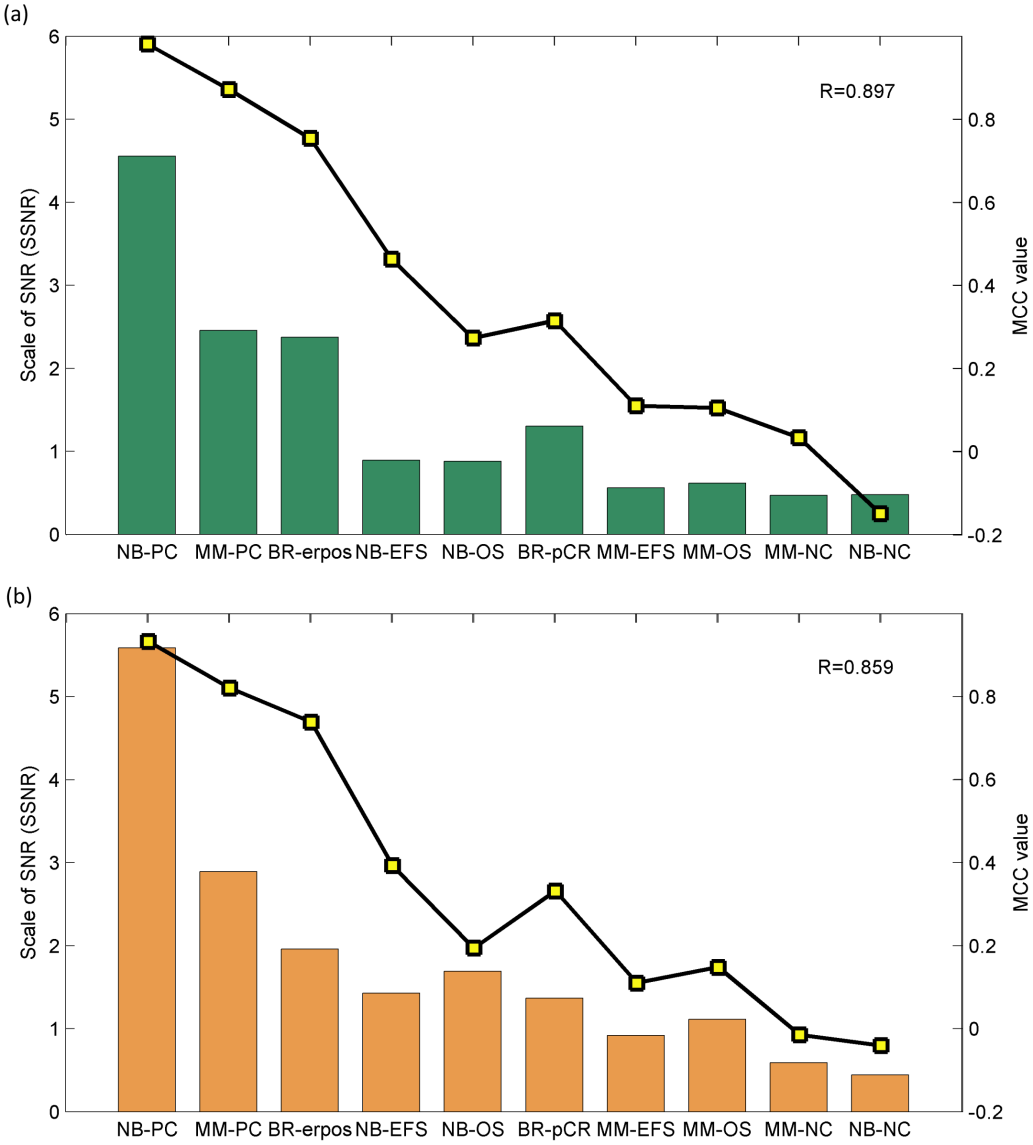
Relationship between SSNR and endpoint predictability based on all training samples. The ex post facto relationship between SSNR values and endpoint predictability (prediction MCC) based on (a) normal and (b) swap modeling using *NCentroid* on all training samples. Here green (a) and orange columns (b) represent the SSNR values obtained from original training and validation sets, while the rectangles faced yellow are corresponding prediction MCC values of models on original validation and training samples, respectively.

The above results showed that the minimum training sample size required for model construction varied with endpoint predictability. Thus it is of vital importance to estimate endpoint complexity in advance of determining the required minimum number of training samples. We proposed an index SSNR in this study, and evaluated its capability as an indication of endpoint predictability. **Figure 3(a)** demonstrated the relationship between SSNR and model performance based on all training samples using *NCentroid*. Here we can see that SSNR correlates well with model performance (MCC values), with a pearson correlation coefficient of 0.897. As a confirmation, we further swapped original training and validation sets, and reevaluated the correlation between SSNR and endpoint predictability. **Figure 3(b)** illustrated corresponding results. A correlation of 0.859 further confirmed that SSNR correlates well with endpoint predictability. Such conclusion was further supported by the correlation of 0.875 and 0.864 for *kNN* and 0.887 and 0.901 for *SVM* classifiers as shown in Figure S3.

### SSNR Guides the Determination of Training Sample Size

The above results confirmed that SSNR was a valid estimation of endpoint predictability and it serves as the basis of training sample size estimation. However, such results were based on ex post facto analysis using all training samples (far more than 60 or 120 ones), leaving it an unaddressed issue whether SSNR could guide training sample size estimation in real applications. Thus we further evaluated the feasibility of using SSNR as a guidance of training sample size estimation from the following two aspects: first, SSNR value was inspected based on 60 or 120 training samples to see if it can successfully differentiate endpoints with different prediction complexities; secondly, the effectiveness of SSNR was verified for estimating required minimum training sample size in real applications using three external validation datasets.

**Figure 4:**
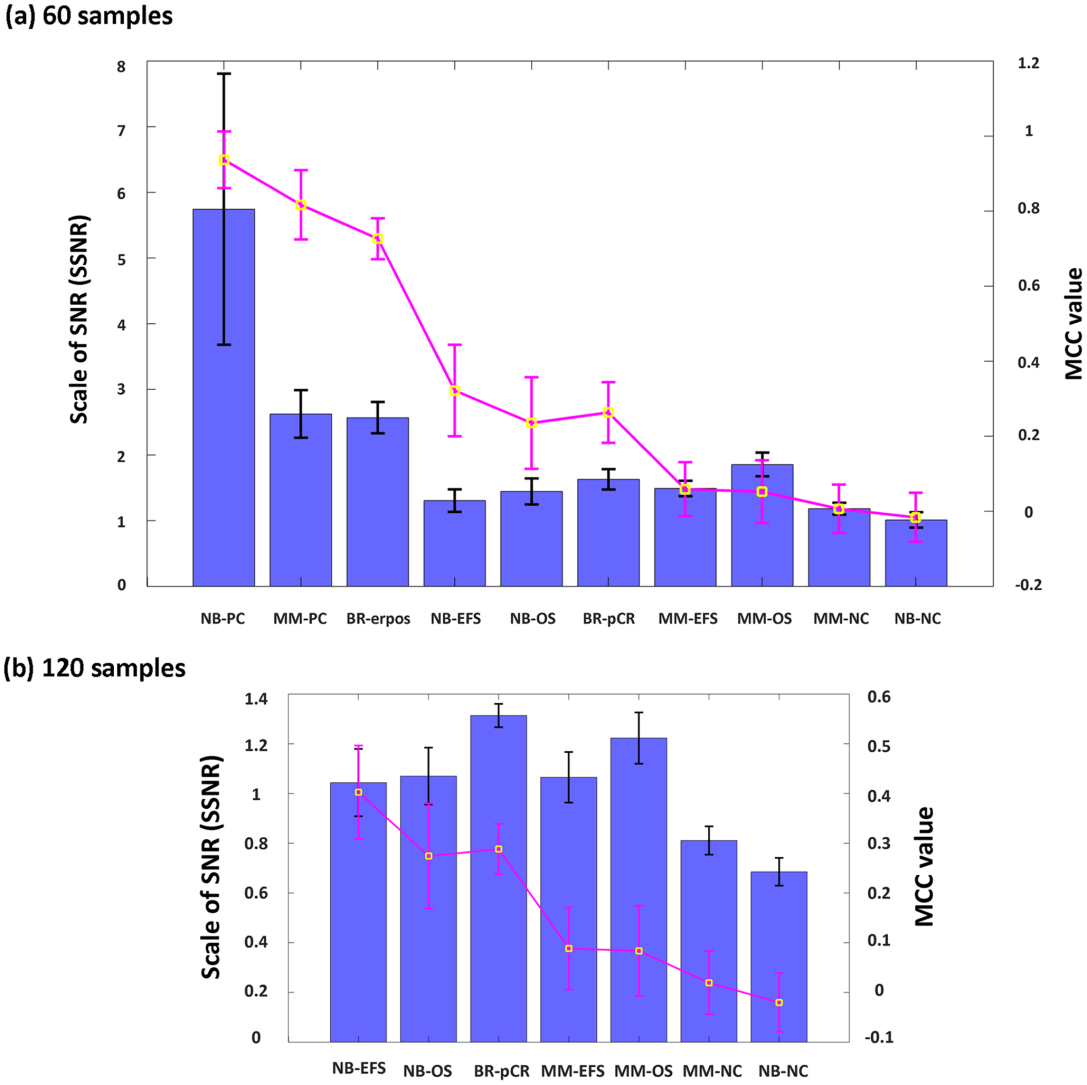
Relationship between SSNR and endpoint predictability based on 60 and 120 training samples. The relationship between SSNR values and endpoint predictability (prediction MCC) based on (a) 60 and (b) 120 training samples using *NCentroid*, respectively. Here blue columns and black bars represent the means and SDs of SSNR values in 100 repetitions, while yellow rectangles and red bars are means and SDs of MCC values.

We randomly retrieved 60 or 120 samples from the original training set, constructed prediction classifiers, predicted original validation sets using the classifier, and then recorded corresponding SSNR and prediction MCC values. To ensure the statistical power, such procedure was repeated 100 times, resulting in 100 pairs of SSNR and MCC values. The capability of SSNR in differentiating endpoints with different complexity was then evaluated from corresponding means and standard deviations (SDs). **Figure 4(a)** demonstrated the relationship between SSNR and MCC values using 60 training samples based on *NCentroid*. We can see that SSNR could successfully differentiate the first three simpler endpoints (SSNR≥2) from others, while no apparent difference was observed among the rest. Excluding the first three endpoints (NB-PC, MM-PC and BR-erpos), we further evaluated the relationship between SSNR and MCC for the rest 7 endpoints using 120 training samples. As shown in **Figure 4(b)**, the five endpoints with SSNR≥1 (NB-EFS, NB-OS, BR-pCR, MM-EFS and MM-OS) were successfully separated from the other two negative controls (SSNR<1) in this case. Therefore, it was confirmed that SSNR could guide training sample size determination efficiently. Corresponding results obtained from *kNN* and *SVM* shown in Figure S4 confirmed the above results.

We further proposed an SSNR-based protocol for training sample size determination in this study. Firstly, 60 training samples were collected and SSNR value was evaluated. If SSNR is larger than 2, 60 training samples size is large enough to achieve a near-optimal prediction model. Otherwise, at least 120 training samples were collected and SSNR value was evaluated again; If SSNR value based on 120 training samples was larger than 1, 120 training samples are enough for model construction this time. Otherwise, the performance of prediction classifier would be deemed as very poor.

**Figure 5:**
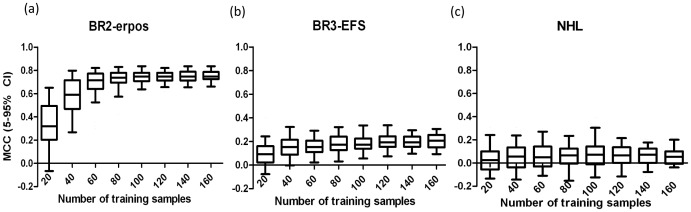
External validation for impact of training sample size. Prediction MCC based on different number of training samples for three external validation datasets.

Three external validation datasets (BR2-erpos, BR3-EFS and NHL) were further used to confirm the performance of abovementioned protocol in real applications. For BR2-erpos, the SSNR value based on 60 training samples (100 repetitions) reached 2.16±0.38 (larger than 2), and thus 60 samples were enough according to the protocol. For BR3-EFS, the SSNR values based on 60 and 120 training samples were 1.55±0.23 (<2) and 1.18±0.11 (>1), respectively. Therefore, 120 training samples were needed to achieve a near-optimal model this time. For NHL, the SSNR values based on 60 and 120 training samples were 1.42±0.22 (<2) and 1.25±0.13 (>1), respectively. As for BR3-EFS, at least 120 training samples were required. **Figure 5(a–c)**, illustrated the performance of prediction classifiers using different number of training samples for above validation datasets. It confirmed the results mentioned above and the capability of the sample size determination protocol proposed in this study.

## Discussion

Microarray data has demonstrated excellent superiority in aiding cancer outcome estimation by providing prediction classifiers. The model reliability relies heavily on the accuracy of statistical parameters estimated from training samples. A small number of training samples cannot provide a highly reliable prediction classifier. Therefore, determining the required minimum number of training samples becomes a vital issue for clinical application of microarrays. Most of current methods are too complex to be utilized for routine application. Therefore, we proposed a simple SSNR-based approach for training sample size determination in this study and illustrated its utility based on three large-scale microarray datasets provided in MAQC-II. The results on three external validation sets confirmed that the SSNR-based protocol was much easier to implement and more efficient for sample size estimation compared to current statistical methods.

Three important findings should be noted in this study. First, it can be seen in **Figure 2** that the number of training samples exerted evident impact on model performance, and the minimum number of training samples required for model construction varied with endpoint predictability. Secondly, SSNR value correlates well with endpoint predictability with a correlation coefficient around 0.9 (**Figure 3**), which implied the possibility of using SSNR as an indication of endpoint predictability. Thirdly, an SSNR-based stepwise function was proposed in this study for determining the minimum number of training samples based on the relationship between training sample size, endpoint predictability, and SSNR value. The discrete relationship between training sample size and complexity of endpoints was also implied by Mukherjee et al. early in 2003 [23], further supporting the SSNR-based determination approach proposed in this study. Moreover, we found that the proposed approach can also be successfully extended to toxicogenomics (see **Figure S5**).

An important aspect of this study is that the confidence of abovementioned findings was also confirmed by both internal and external validation strategies. For internal validation, two positive (NB-PC, MM-PC) and two negative control (NB-NC, MM-NC) datasets were essential to assess the performance of clinically relevant endpoints against the theoretical maximum and minimum performance provided by the controls. Specifically, the much higher SSNR values for two positive control datasets shown in **Figure 4(a)** confirmed the capability of using SSNR as an indication of endpoint predictability, while the negligible impact of training sample size on model performance in two negative control datasets further precludes the possibility of obtaining false positive results. Thus, including positive and negative control datasets in such analyses would be of great help in ensuring the reliability of the final results. Moreover, the reliability of a training process can only be ascertained by external validation samples. Therefore, the external validation datasets together with internal controls have played an important role in confirming the capability of SSNR-based training sample size determination approach in this study.

Similar results obtained from three well-known classification methods used in this study (i.e. *NCentroid*, *kNN and SVM*, with corresponding results provided in **Figure 2** and **Figure S1 and S2**, respectively) further confirmed the reliability of the SSNR-based training sample size estimation approach. The reason is out of the scope of this study. However, this phenomenon conforms to the lack of significant differences among a large number of classification methods reported for microarray applications in terms of prediction performance [24]. A similar conclusion was also proposed by MAQC-II [6]. Such results would preclude the restriction of different classification algorithms, and further extend the applicability of the SSNR-based training sample size determination approach.

The superiority and applicability of the SSNR-based approach can be summarized as follows. Firstly, from a statistical point of view, it was not biased by deduction procedures by avoiding sophisticated statistical calculations. Secondly, in respect of clinical routine applications, it is much more straightforward and efficient, as the only requirements are collecting 60 and/or 120 samples and calculating corresponding SSNR values. In the meantime, the SSNR-based protocol can also provide a valuable strategy for estimating the performance of classifiers in advance. Taking external validation datasets shown in **Figure 5** as an example, SSNR values being 2.16±0.38, and 1.18±0.11 for BR2-erpos, and BR3-EFS also implied that the performance of final prediction classifiers in this case would be excellent, and moderate, respectively.

### Conclusions

Microarray technology combined with pattern recognition has been demonstrated as a promising strategy in providing prediction classifiers for cancer diagnosis, prognosis and treatment response estimation and so on. Compared to traditional experience-based diagnosis relying on complex biochemical testing and miscellaneous image systems, microarray-based prediction classifiers, if reliably constructed from enough training samples, would provide a much more objective, accurate, and valid depiction of cancer outcomes. Consequently, the SSNR-based training sample size determination approach would provide great convenience for clinical application of microarrays in cancer outcome assessment by providing a simple and pragmatic way of estimating training sample size. Moreover, the fact that training sample size impacts the performance of final prediction classifiers further implied the importance of systematically evaluating each procedure in the model construction process and developing practical guidance for microarray-based class comparison analysis.

## Supporting Information

Figure S1 **An additional figure for the** **impact of training sample size using** ***kNN*** **.** Prediction MCC based on different number of training samples for 10 endpoints using *kNN*.
(TIF)

Figure S2 **An additional figure for the impact of training sample size using** ***SVM*** **.** Prediction MCC based on different number of training samples for 10 endpoints using *SVM*.
(TIF)

Figure S3 **An additional figure for the relationship between SSNR and endpoint predictability based on all training samples.** The ex post facto relationship between SSNR values and endpoint predictability (prediction MCC) based on normal and swap modeling using *kNN* and *SVM* on all training samples.
(TIF)

Figure S4 **An additional figure for the relationship between SSNR and endpoint predictability based on 60 and 120 training samples.** The relationship between SSNR values and endpoint predictability (prediction MCC) based on (a) 60 and (b) 120 training samples using *kNN* and *SVM*, respectively.
(TIF)

Figure S5 **An additional figure for the impact of training sample size for toxicogenomic dataset NIEHS.**
(TIF)

Table S1 Corresponding ν values for different training sample size of 10 endpoints using *NCentroid.*
(DOCX)

Methods S1. (DOC)
