# Supplementary figures and images for: Determination of Minimum Training Sample Size for Microarray-Based Cancer Outcome Prediction–An Empirical Assessment
Source: PLoS One. 2013 Jul 5;8(7):e68579. doi: 10.1371/journal.pone.0068579 (PMC3702597; doi:10.1371/journal.pone.0068579)

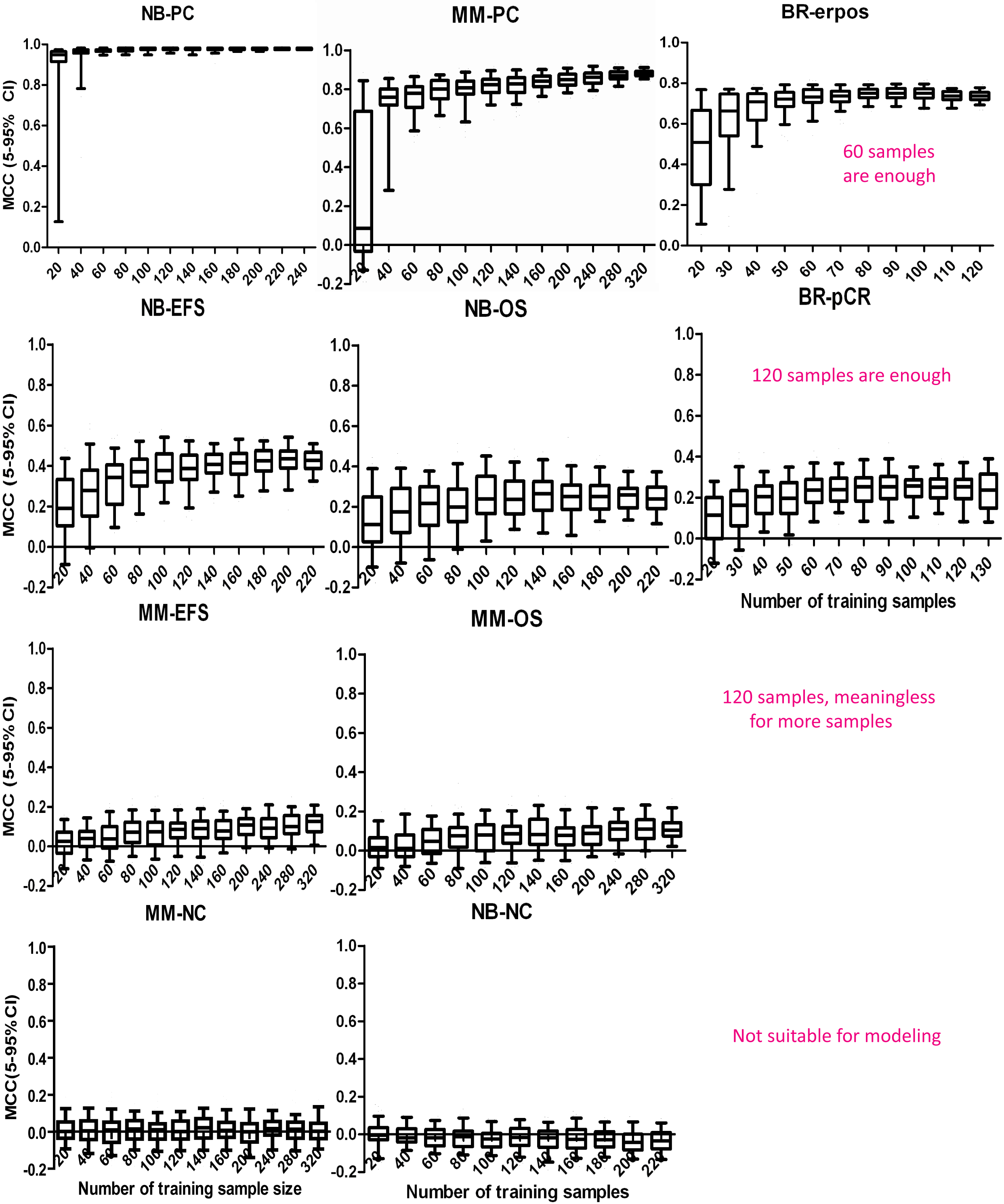

Supplement: Figure S1 — An additional figure for the impact of training sample size using kNN . Prediction MCC based on different number of training samples for 10 endpoints using kNN. (TIF) [file pone.0068579.s001.tif]

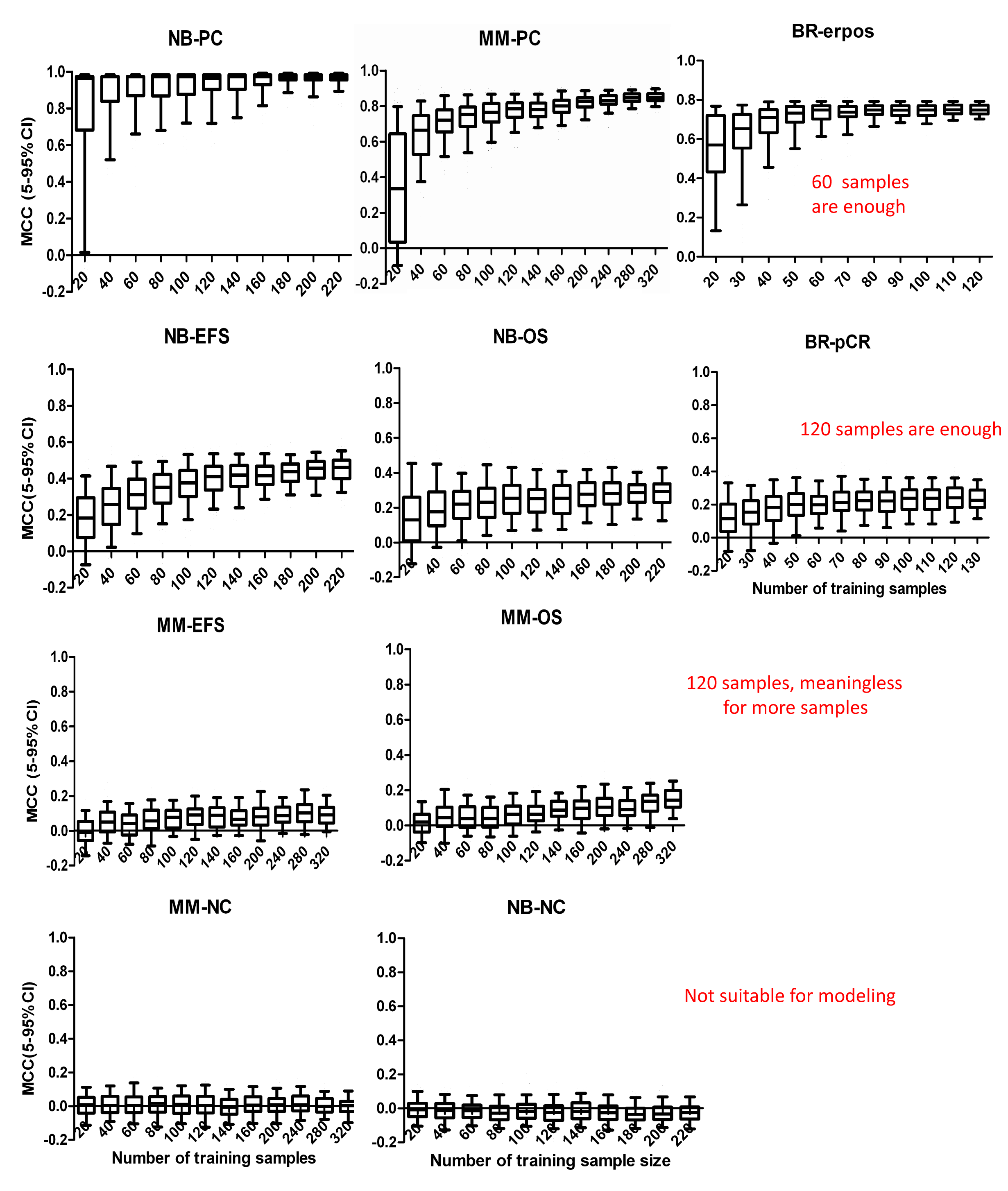

Supplement: Figure S2 — An additional figure for the impact of training sample size using SVM . Prediction MCC based on different number of training samples for 10 endpoints using SVM. (TIF) [file pone.0068579.s002.tif]

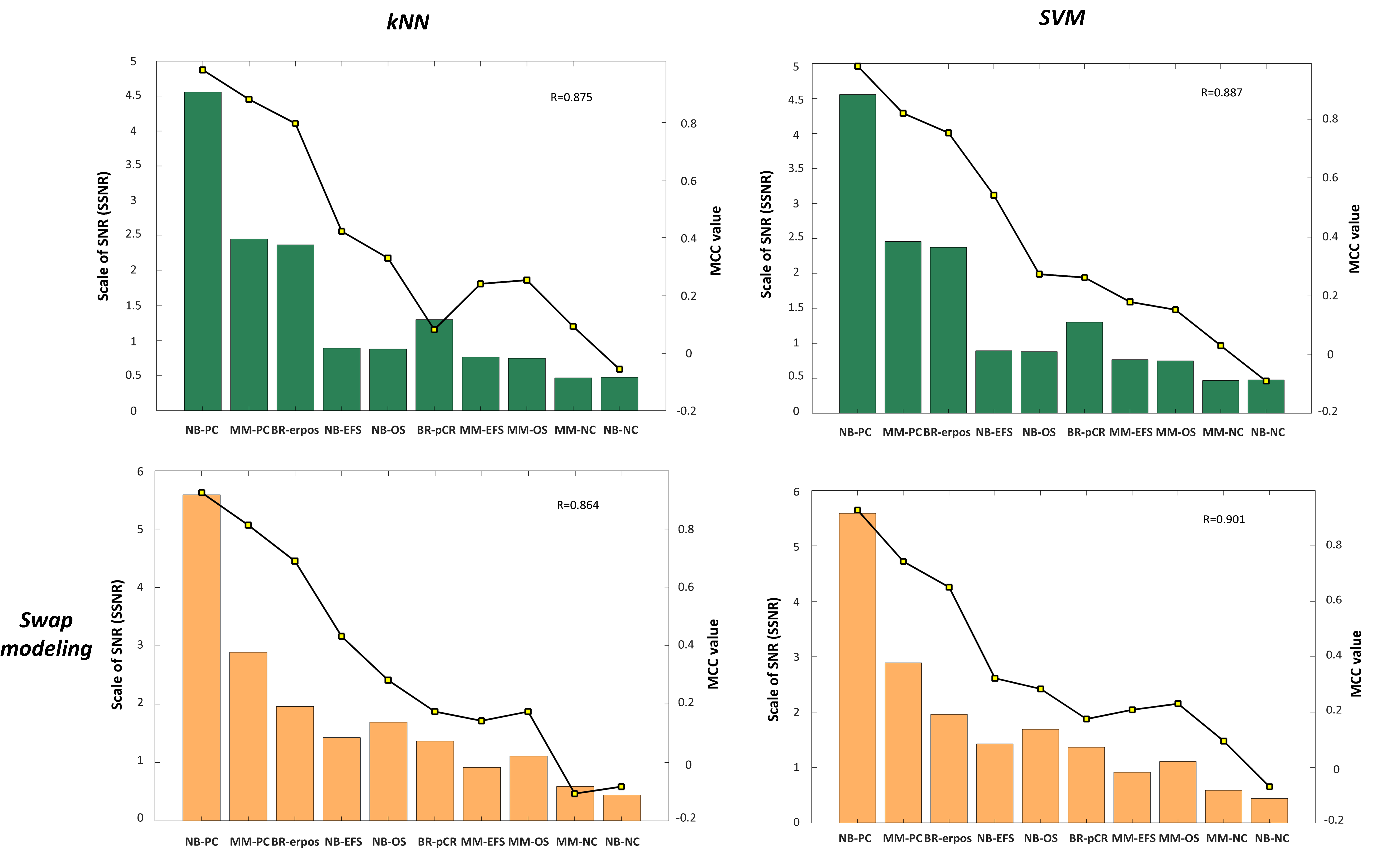

Supplement: Figure S3 — An additional figure for the relationship between SSNR and endpoint predictability based on all training samples. The ex post facto relationship between SSNR values and endpoint predictability (prediction MCC) based on normal and swap modeling using kNN and SVM on all training samples. (TIF) [file pone.0068579.s003.tif]

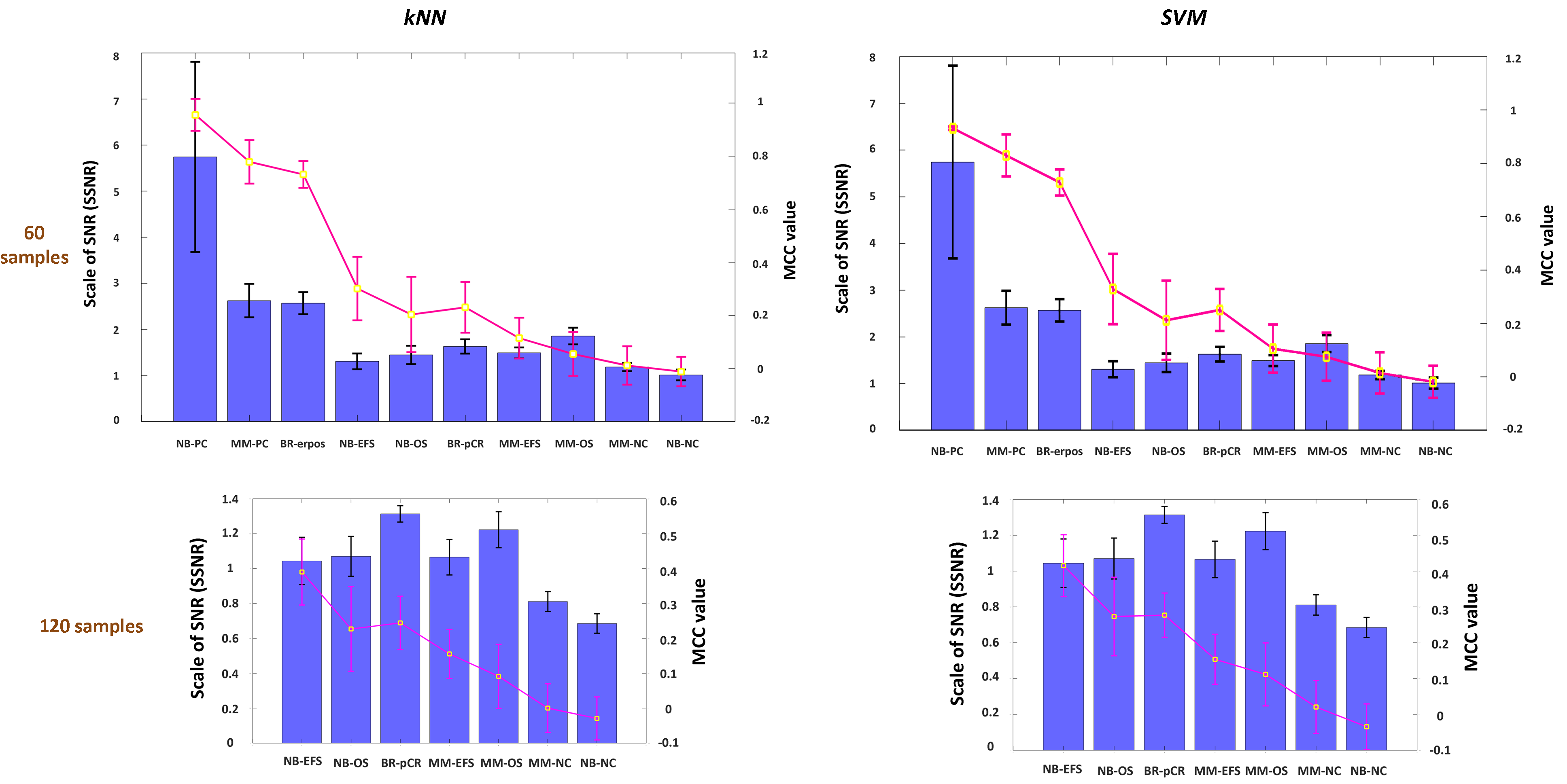

Supplement: Figure S4 — An additional figure for the relationship between SSNR and endpoint predictability based on 60 and 120 training samples. The relationship between SSNR values and endpoint predictability (prediction MCC) based on (a) 60 and (b) 120 training samples using kNN and SVM, respectively. (TIF) [file pone.0068579.s004.tif]

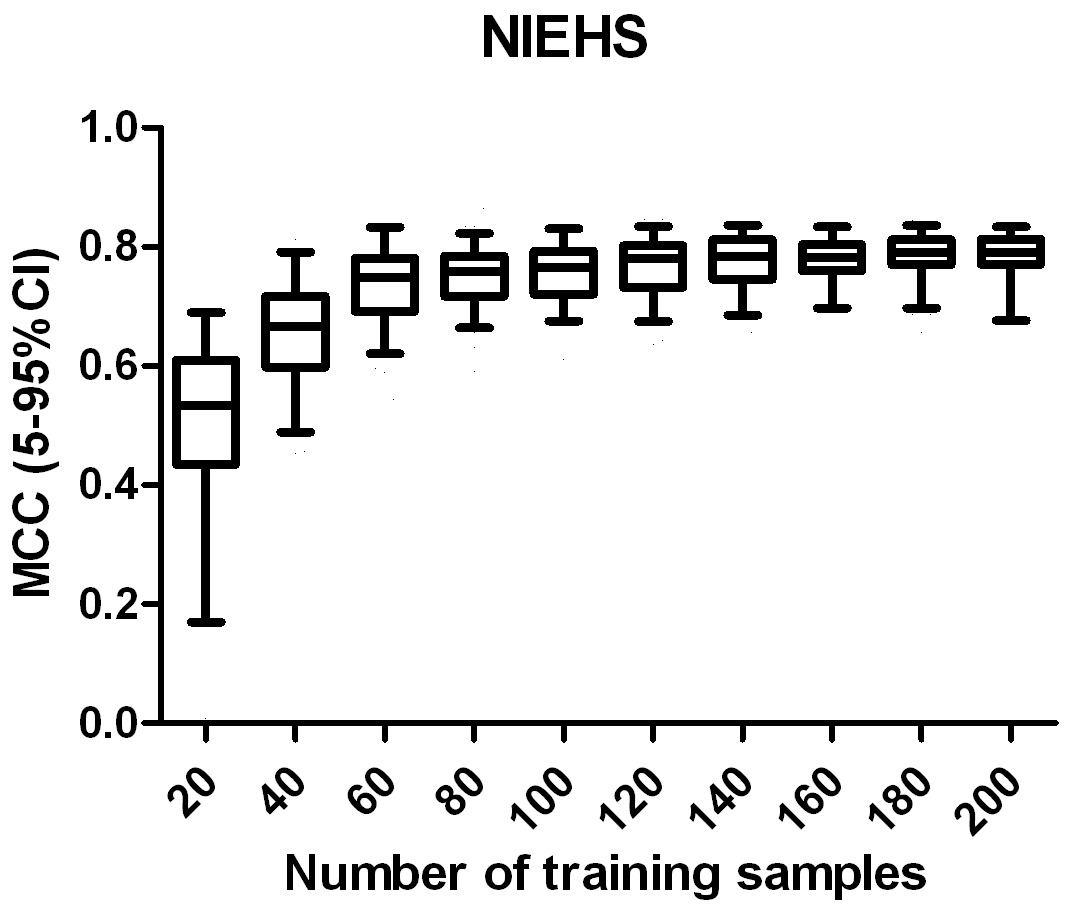

Supplement: Figure S5 — An additional figure for the impact of training sample size for toxicogenomic dataset NIEHS. (TIF) [file pone.0068579.s005.tif]
