# Supplementary material for: Determination of Minimum Training Sample Size for Microarray-Based Cancer Outcome Prediction–An Empirical Assessment
Source: PLoS One. 2013 Jul 5;8(7):e68579. doi: 10.1371/journal.pone.0068579 (PMC3702597; doi:10.1371/journal.pone.0068579)
Supplement: Table S1 — Corresponding ν values for different training sample size of 10 endpoints using NCentroid. (DOCX) [file pone.0068579.s006.docx]

**Table S1**. Corresponding $v$ values for different training sample size of 10 endpoints using *NCentroid*

| **Sample size** | **NB-PC** | **MM-PC** | **BR-erpos** | **NB-EFS** | **NB-OS** | **BR-pCR** | **MM-EFS** | **MM-OS** | **MM-NC** | **NB-NC** |
| --- | --- | --- | --- | --- | --- | --- | --- | --- | --- | --- |
| 40 | 5.653 | 32.194 | 15.594 | 27.061 | 27.966 | 31.882 | 246.503 | 11.122 | 281.992 | -9.727 |
| 60 | 0.331 | 1.866 | 1.536 | 1.893 | 6.418 | 8.879 | 16.043 | 22.642 | -14.801 | -1.247 |
| 80 | 0.379 | 0.621 | 0.380 | 1.858 | 0.593 | 0.186 | 2.665 | 7.193 | 4.677 | 10.271 |
| 100 | 0.049 | -0.083 | -0.072 | 0.978 | 0.959 | 0.161 | -0.946 | 0.242 | -1.653 | 2.839 |
| 120 | 0.115 | 0.244 | 0.040 | 0.440 | 0.452 | 0.696 | 3.993 | 0.953 | 16.233 | -3.741 |
| 140 | 0.018 | 0.020 |  | 0.379 | -0.056 |  | -0.474 | -0.104 | -4.071 | 2.133 |
| 160 | 0.008 | 0.077 |  | 0.238 | 0.619 |  | 1.439 | 0.216 | -8.087 | 5.025 |
| 180 | 0.019 |  |  | 0.000 | 0.010 |  |  |  |  | -0.745 |
| 200 | -0.003 | 0.015 |  | 0.307 | -0.144 |  | 0.222 | 1.531 | -23.411 | 0.863 |
| 220 | 0.016 |  |  | -0.072 | -0.424 |  |  |  |  | 0.500 |
| 240 |  | 0.044 |  |  |  |  | 0.189 | 0.175 | 6.675 |  |
| 260 |  |  |  |  |  |  |  |  |  |  |
| 280 |  | 0.020 |  |  |  |  | 0.763 | -0.471 | -7.053 |  |
| 300 |  |  |  |  |  |  |  |  |  |  |
| 320 |  | 0.014 |  |  |  |  | -0.218 | 0.148 | 13.272 |  |
