# Supplementary material for: Determination of Minimum Training Sample Size for Microarray-Based Cancer Outcome Prediction–An Empirical Assessment
Source: PLoS One. 2013 Jul 5;8(7):e68579. doi: 10.1371/journal.pone.0068579 (PMC3702597; doi:10.1371/journal.pone.0068579)
Supplement: Methods S1. — (DOC) [file pone.0068579.s007.doc]

**Supplementary Methods**

Details about the model construction procedure are described as follows (according to the footnotes in the Figure):


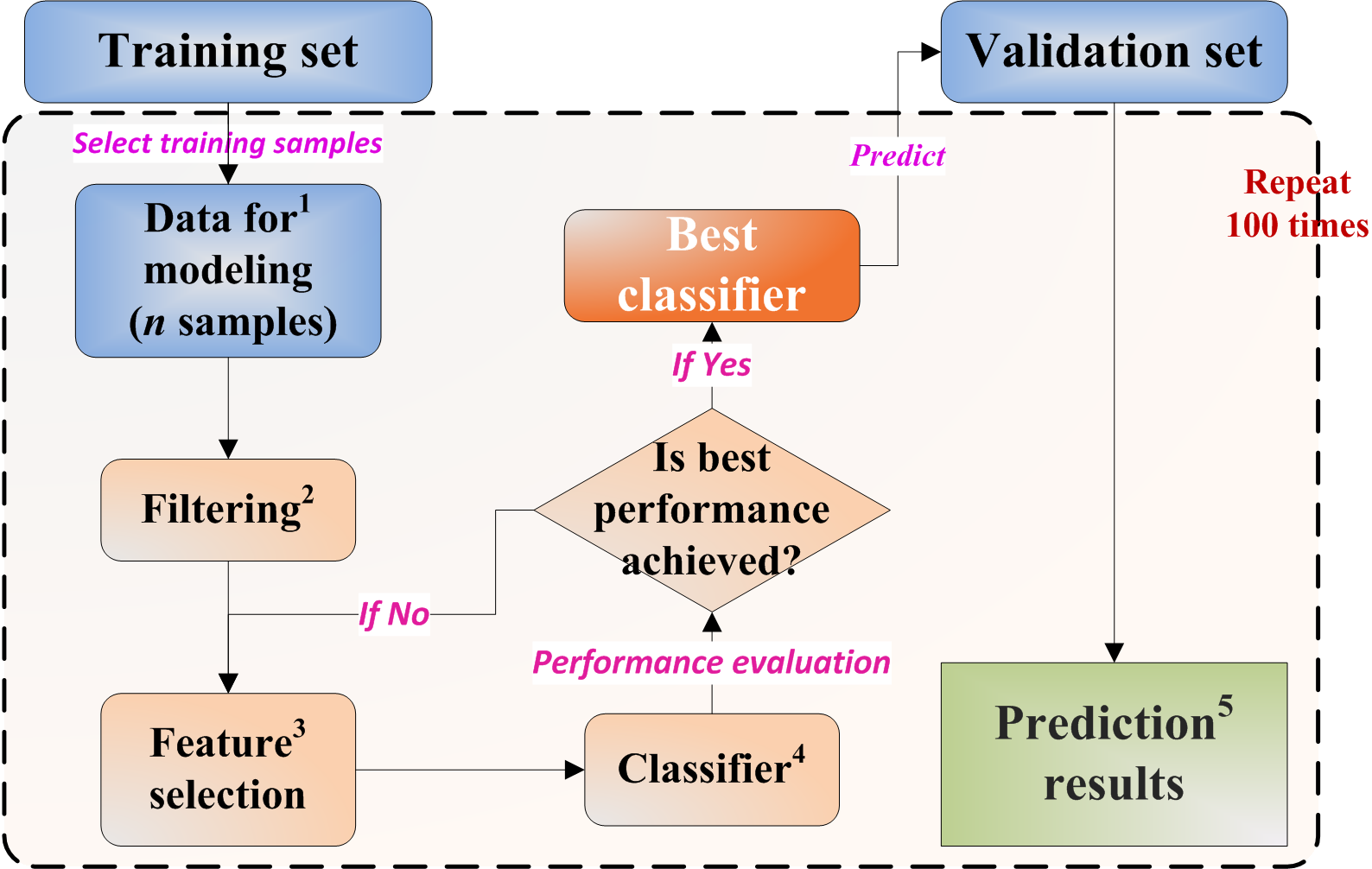


(1) Dataset generation – dataset with a specific sample size was firstly retrieved from the original training set as new training samples.

(2) Feature filtering – This step was employed to generate an initial pool of features for subsequent analyses. The features were firstly rank-ordered according to the absolute values of signal-to-noise ratio, and the top 100 ones were then retained for further analyses.

(3) Feature selection – A sequential forward feature selection method was applied. Concisely, features associated with the best performance were added into the model step by step. Here the performance of the resulting classifier was evaluated using 5-fold cross-validation (10 repetitions) and the prediction accuracy was recorded.

(4) Classifier selection – For classifier *i* (*i* is the number of features used in the classifier), if the subsequent five classifiers performed worse than or equal to that of classifier *i*, the procedure stopped and the *i*th classifier was selected as the best classifier. Otherwise, steps 3 and 4 were repeated.

(5) Prediction – The best classifier was utilized to predict original validation samples, and corresponding prediction results were recorded.

Since the sample splitting (step 1) was random in nature, the above process was repeated 100 times, thereby generating 100 best classifiers. The results of the 100 classifiers were utilized as an indication of model performance corresponding to specific training sample size.
